# Editorial: The role and mechanism of metabolic dysfunction in the development of neurodegenerative disease

**DOI:** 10.3389/fnmol.2024.1373532

**Published:** 2024-02-12

**Authors:** Samuel T. Henderson

**Affiliations:** Cerecin Inc, Denver, CO, United States

**Keywords:** metabolism, Alzheimer's, BDNF, western diet, miRNA, astrocyte, glutamate

The intersection of metabolic dysfunction and neurodegenerative diseases has emerged as a critical area of study. With an aging global population, understanding the underlying mechanisms that contribute to these diseases is more important than ever. In this Research Topic, three original research articles and an excellent review each contribute distinct insights into how metabolic processes influence the development and progression of Alzheimer's disease and other disorders. These studies identify; the causes, potential genetic risk factors, outcomes, and potential biomarkers of metabolic dysfunction that are associated with neurodegenerative diseases.

Kelty et al. provide compelling evidence linking diet-induced obesity to neurodegenerative disorders through mitochondrial dysfunction. Focusing on the effects of a Western diet (WD), characterized by high fat and fructose, the study found significant reductions in mitochondrial respiration in key brain regions including the hippocampus and prefrontal cortex (PFC). Ossabaw swine were fed a WD for 6-months. The WD was defined as comprising of 40.8% Kcal from carbohydrates, 16.2% Kcal from protein, 42.9% kCal from fat, and 2% Kcal from cholesterol. Using high resolution respirometry measurements from isolated brain mitochondria, WD feeding resulted in reduced State 3 respiration in Complex I and II, reduced uncoupled mitochondrial respiration in the hippocampus and PFC, reduced protein levels of oxidative phosphorylation of Complexes I–V in the PFC, and significantly increased markers of antioxidant defense in the hippocampi and PFC. These findings are crucial as they highlight the potential of dietary factors in exacerbating neurodegenerative processes, suggesting that metabolic health is intricately linked to neurodegenerative disease risk. This study adds to the growing body of evidence that lifestyle factors, including diet, significantly impact the risk and progression of neurodegenerative diseases.

de Assis et al. examined the impact of the Val66Met polymorphism in the Brain-derived neurotrophic factor (BDNF) gene on BDNF expression in human muscle under metabolic stress. In humans, a single nucleotide polymorphism in BDNF has been identified in ~20% of the population that results in a Valine to Methionine substitution in the 66th amino-acid position of the synthesized protein and is referred to as “Val66Met”. This polymorphism has been implicated in various neurological diseases and this study examined a link between metabolic stress and this polymorphism. Thirteen male recreational athletes were recruited into the study and muscle biopsies were obtained before and immediately following a VO2max test. The results indicated that BDNF expression levels were influenced by the genotype according to the presence of the polymorphism. BDNF expression was 1.3-fold lower in carriers of the Met66 allele than that from the Val66 carriers, and BDNF expression levels decreased by an average of 1.8-fold following the VO2max test, regardless of the individual's genotype. The results of this study indicate that metabolic stress downregulates BDNF expression but not plasma BDNF concentrations. These findings emphasize the role of genetic factors in metabolic responses and their potential influence on neurodegenerative disease progression. The study contributes to the growing understanding of how genetic variations can influence metabolic processes and potentially affect the risk and progression of neurodegenerative diseases.

Fan et al. delved into metabolic changes in astrocytes in Alzheimer's disease (AD). Astrocytes are crucial for maintaining brain homeostasis and show significant metabolic alterations in AD, including the depletion of essential metabolites and impaired metabolic fluxes. The study used single-nucleus transcriptomics of individual astrocytes in healthy and pathological cells at different stages of AD in the PFC. The snRNA-seq datasets were downloaded from the NCBI Gene Expression Omnibus database. Changes in important metabolites, such as 2-oxoglutarate (2OG), acetyl-coenzyme A (CoA), aspartate, pyruvate, and glucose-6-phosphate (G6P) were noted in the study. Higher levels of glutamate and lower levels of glutamine and 2OG were found in AD population suggesting downregulation of GLUL and GLUD1. These alterations in astrocyte metabolism, particularly in glutamate metabolism, provide crucial insights into AD's pathogenesis. The study suggests that targeting these metabolic abnormalities could be a novel approach to managing AD. This research contributes significantly to our understanding of the cellular mechanisms underlying neurodegenerative diseases and underscores the importance of metabolic processes in maintaining neuronal health.

Karuga et al. reviewed the role of microRNAs (miRNAs) in obstructive sleep apnea (OSA) and its associated metabolic complications. OSA, a common respiratory disorder, has been linked to various metabolic issues, including diabetes and metabolic syndrome. The study identified specific miRNAs as potential biomarkers for OSA and its metabolic complications, providing a foundation for understanding the epigenetic regulation of metabolic disturbances in OSA. The authors highlighted that OSA alters the expression of specific miRNAs. They conclude that the most important examples include miRNA-181a and miRNA-199a. Both of these miRNAs play an important role in the metabolic consequences of OSA development. This research opens new avenues for diagnosis and treatment, emphasizing the role of epigenetic mechanisms in the interplay between respiratory dysfunction and metabolic health.

The convergence of findings from these studies highlights metabolic dysfunction as a central player in neurodegenerative diseases, an idea with growing support as described by Cunnane et al. (2020). They collectively suggest that diet and lifestyle factors can influence mitochondrial function and contribute to development of neurodegenerative diseases, there exists potential genetic predispositions in metabolic responses such as polymorphisms in BDNF, adds to the growing body of literature identifying metabolic alterations in AD at the single astrocyte level, and identify potential biomarkers to aid in diagnosis and treatment of these diseases. The intersection of these studies on the theme of metabolic dysfunction in neurodegeneration underscores the necessity of a comprehensive approach to understanding and managing these complex diseases. By integrating insights from dietary, genetic, cellular, and epigenetic perspectives, we can better comprehend and address the multifaceted nature of neurodegenerative disorders. Future research in this domain holds the promise of novel therapeutic strategies, improved diagnostic tools, and a deeper understanding of the mechanisms underlying these debilitating diseases. The implications of these studies are far-reaching, suggesting that interventions targeting metabolic health could significantly impact the prevention and management of these diseases.

Furthermore, the research emphasizes the need for a multidisciplinary approach that combines insights from nutrition, genetics, cellular biology, and epigenetics. This approach could lead to more effective treatments and preventative measures that are tailored to individual risk profiles and disease mechanisms. As we continue to unravel the complex interplay between metabolic dysfunction and neurodegenerative diseases, it is likely that new pathways for intervention and management will emerge.

This editorial brings to the forefront the crucial role of metabolic dysfunction in neurodegenerative diseases and paves the way for future research and therapeutic developments. It encourages a holistic view of these conditions, integrating various aspects of health and disease, from genetics and cellular biology to lifestyle and environmental factors.

## Author contributions

SH: Writing – original draft, Writing – review & editing.
